# Comparison of Different Machine Models Based on Contrast-Enhanced Computed Tomography Radiomic Features to Differentiate High From Low Grade Clear Cell Renal Cell Carcinomas

**DOI:** 10.3389/fonc.2021.659969

**Published:** 2021-05-26

**Authors:** Xu Pei, Ping Wang, Jia-Liang Ren, Xiao-Ping Yin, Lu-Yao Ma, Yun Wang, Xi Ma, Bu-Lang Gao

**Affiliations:** ^1^ CT/MRI Room, Affiliated Hospital of Hebei University, Baoding, China; ^2^ Department of Pharmaceutical Diagnostics, GE Healthcare China (Shanghai) Co Ltd., Shanghai, China; ^3^ Key Laboratory of Cancer Radiotherapy and Chemotherapy Mechanism and Regulations, Baoding, China

**Keywords:** renal clear cell carcinoma, enhanced computed tomography, imaging histology, logistic regression, radiomics

## Abstract

**Purpose:**

This study was to investigate the role of different radiomics models with enhanced computed tomography (CT) scan in differentiating low from high grade renal clear cell carcinomas.

**Materials and Methods:**

CT data of 190 cases with pathologically confirmed renal cell carcinomas were collected and divided into the training set and testing set according to different time periods, with 122 cases in the training set and 68 cases in the testing set. The region of interest (ROI) was delineated layer by layer.

**Results:**

A total of 402 radiomics features were extracted for analysis. Six of the radiomic parameters were deemed very valuable by univariate analysis, rank sum test, LASSO cross validation and correlation analysis. From these six features, multivariate logistic regression model, support vector machine (SVM), and decision tree model were established for analysis. The performance of each model was evaluated by AUC value on the ROC curve and decision curve analysis (DCA). Among the three prediction models, the SVM model showed a high predictive efficiency. The AUC values of the training set and the testing set were 0.84 and 0.83, respectively, which were significantly higher than those of the decision tree model and the multivariate logistic regression model. The DCA revealed a better predictive performance in the SVM model that possessed the highest degree of coincidence.

**Conclusion:**

Radiomics analysis using the SVM radiomics model has highly efficiency in discriminating high- and low-grade clear cell renal cell carcinomas.

## Introduction

Clear cell renal cell carcinoma (ccRCC) accounts for 70% of renal cancers (1). Since the long-term survival of clear cell carcinoma patients correlates negatively to the Fuhrman grading (2–4), it is crucial to accurately grade clear cell carcinoma of the kidney as early as possible. Grading ccRCC through aspiration biopsy is controversial as the operation itself carries risk of metastatic spread (5, 6). Previous studies on RCC were mostly based on analysis of images of conventional computed tomography (CT) (7–9), which was often interfered by human factors and lack of quantification. Through precise quantitative analysis of medical images, radiomics provides researchers an effective way to detect biological characteristic changes caused by tumor microenvironment (10–12). Classic CT information or CT-based radiomics has been applied to establish predictive models for ccRCC grade. In three logistic regression models of radiomics based on non-texture features, texture fraction and non-texture feature combined with texture fraction for identifying high- and low-grade ccRCCs (13), the area under the operating curve (AUC) values in the three models were 0.826, 0.878, and 0.843 for the training set and 0.671, 0.771, and 0.780 for the testing set, respectively. Some image features like tumor size (TS) and permeability surface-area product (PS) were helpful in differentiating high- from low-grade ccRCCs based on conventional CT studies, with the AUC of TS and PS of 0.7 (14). The sensitivity and specificity were 0.8 and 0.6 for TS and 0.7 and 0.8 for PS, respectively. Moreover, gene fragments and radiomics can be combined to establish a two-group model for differentiating ccRCC from non-clear cell RCC (non-ccRCC), with the AUC of the training set and testing set being 0.969 and 0.900, respectively (15). Some studies confirmed that necrosis can independently predict the biological invasiveness of ccRCCs (16, 17). Moreover, only the logistic regression model was utilized in most of these studies lacking comparison between different predictive modeling methods. Therefore, in this study, three models including logistic regression, decision tree and support vector machine (SVM) were established and compared for ccRCC grading performance.

## Materials And Methods

### Patients

**Figure 1:**
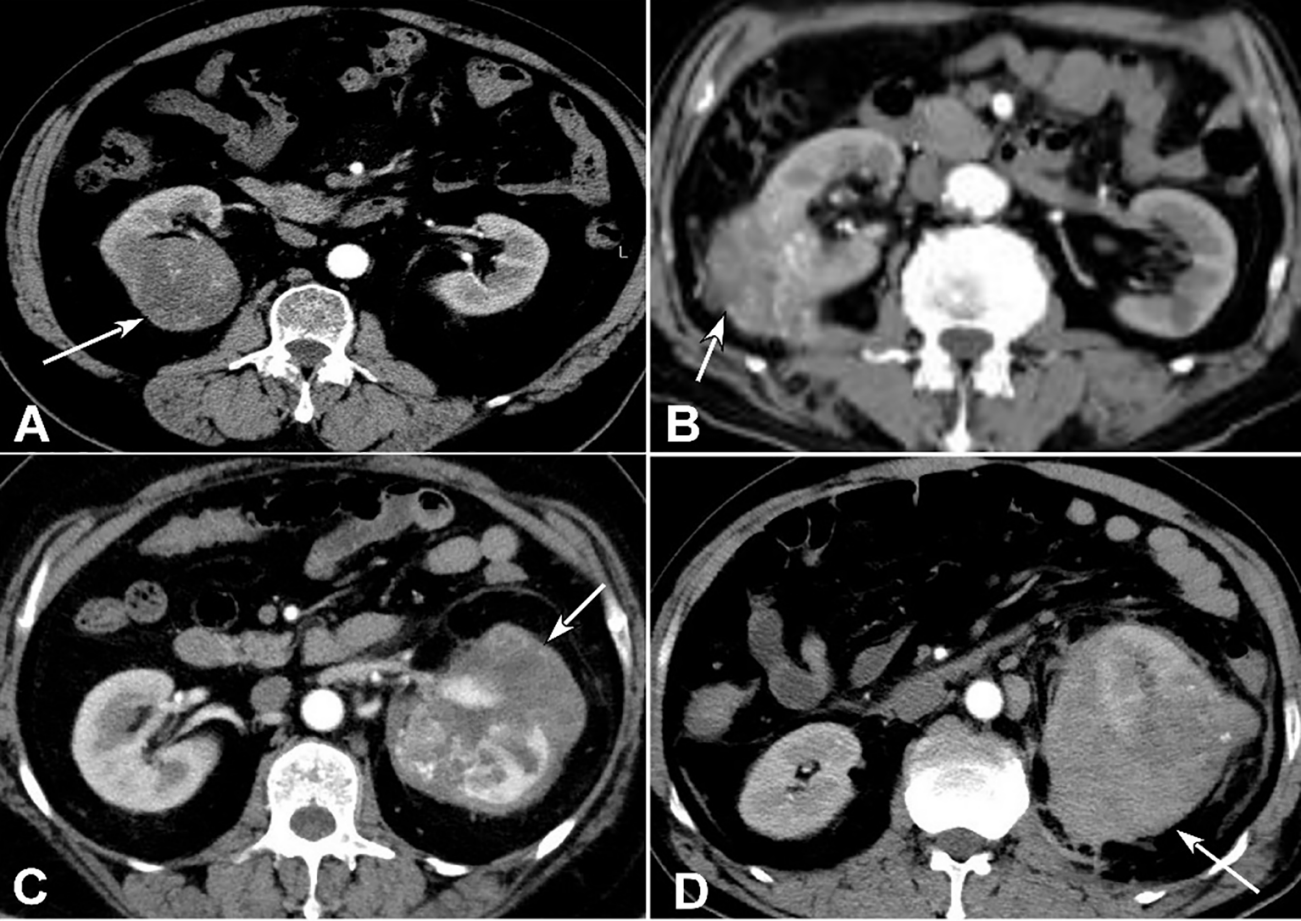
Clear cell renal cell carcinomas (ccRCC) with different grades. **(A)** Grade I ccRCC (arrow) was demonstrated. **(B)** Grade II ccRCC was shown (arrow). **(C)** Grade III ccRCC was revealed (arrow). **(D)** Grade IV ccRCC (arrow) was displayed.

This retrospective study was approved by the Ethics Review Committee of Affiliated Hospital of Hebei University with all patients given their signed informed consent. All methods were performed in accordance with the relevant guidelines and regulations. Patients with ccRCC were enrolled between January, 2017 and December, 2018 in our hospital. Inclusion criteria were a single lesion with clear grades of RCC and preoperative enhanced CT images in the cortical phase with fast-in and fast-out enhancement (cortical phase showed the clearest). Exclusion criteria were: (I) carcinomar metastasis, (II) cystic changes in the lesion of carcinoma, (III) necrosis volume >80% of the maximal lesion volume, and (IV) poor image quality. In accord with these criteria, 42 unqualified samples were excluded, and 190 eligible samples were included. In this study, I-II grade ccRCC was defined as low-grade renal clear cell carcinoma, and III-IV grade ccRCC was defined as high-grade renal carcinoma (18) (**Figure 1**). Among the qualified 190 patients with ccRCC, 133 cases were of grade I-II ccRCC and 57 cases were of grade III-IV ccRCC, including 98 males and 92 females with an age range of 27–88 years (mean 58.30 ± 8.70) (**Table 1**). Their maximal diameters of the carcinoma ranged 2-12 cm (mean 5.6 ± 4.4) from post-operative pathological exams.

**Table 1:** Demography of patients in two sets.

Variables	Training set	Testing set
Case no.	122(64%)	68 (36%)
Sex		
Male	66(54%)	33(49%)
Female	56(46%)	35(51%)
Age mean (range, y)	55.6(28-85)	56.1(31-87)
< 60	59(48%)	35(51%)
≧60	33(49%)	63(52%)
Subtype		
Low-grade ccRCC	81(66%)	52(76%)
High-grade ccRCC	41(34%)	16(24%)
Tumor size (cm, mean ± SD)		
Low-grade ccRCC	6.48 ± 3.46	6.57 ± 3.31
High-grade ccRCC	7.21 ± 3.13	8.31 ± 3.31

Low grade, grades I-II; High-grade, grades III-IV; SD, standard deviation.

### CT Image Acquisition

Abdominal plain and enhancement CT scans were performed with a 64-row CT scanner (GE Discovery HD 750, GE Health Care, Chicago, IL, USA). Contrast agent was iodophor alcohol, a non-ionic iodine contrast agent. The post-injection scanning time points were 30-35s, 50-60s and 180s, covering the medullary phase and renal pelvis stage. Scanning parameters were as follows: cortical phase, pitch: 0.984:1, layer thickness: 5 mm, field of view: 40 cm×40 cm, matrix: 512×512, tube voltage: 100-120 kV, tube current: 134-409 mA, window width: 250-450 HU, and window position: 30-50 HU.

### Volumes of Interest (VOIs) Segmentation

The cortical phase images of enhanced CT from 190 subjects were imported into the ITK-SNAP software (19), and the region of interest (ROI) was delineated by one radiologist with 8 years of working experience and checked by another radiologist with 10 years of working experience.

### Radiomics Feature Extraction and Selection

The radiomics features were extracted from the original and filtered images with the AK software (Artificial Intelligence Kit V3.0.0.R, GE Healthcare, China). A total of 402 features were obtained, including 42 histogram features, 144 gray-level co-occurrence matrices features (GLCM), 180 gray-level run length matrices features (GLRM), 11 gray-level zone matrices features (GLSZM), 15 shape-based features, and 10 Haralick features. The feature selection procedure was as follows: Firstly, the data of patients from January 2017 to April 2018 were included in the training set, and the data of patients from April 2018 to December 2018 were included in the testing set, with the data of 122 patients in the training set (with 81 cases of I-II ccRCC and 41 cases of III-IV ccRCC) and 68 patients in the testing set (with 52 cases of I-II ccRCC and 16 cases of III-IV ccRCC). Secondly, the data were preprocessed, including replacing missing values with the median value and standardizing the Z-score of features in all data. Thirdly, the extracted features were analyzed by one-way ANOVA and Wilcox rank-sum test, with the significant P value set at less than 0.05. Then, the least absolute shrinkage and selection operator (LASSO) method, which has been shown to be suitable for high dimensional data analysis (13), were used for further feature screening. The LASSO method selects features using a tuning parameter (Lambda), with some coefficients in the covariance can be shrunk to zero when the cross-validation error is the smallest. All the feature selection procedure performed on the training set and applied on the testing set. The finally selected features were used to construct models.

## Radiomic Model Building and Validation

The ROC curves of each model in the training set (data of 122 patients) and testing set (68 patients) were calculated with all available patients and the AUC values were derived (**Figure 3**). The predictive performances of three models (logistic regression, decision tree, and SVM) were compared for analysis. The decision curve analysis (DCA) was conducted to evaluate the clinical usefulness of the models for ccRCC prediction. DCA quantified the net benefits at different threshold probabilities in the training and testing set (**Figure 4**).

### Statistical Analysis

Statistical analysis was performed with the R software (version: 3.6.3, www.r-project.org). The Chi-square test was used to evaluate the distribution difference in high and low-grade cc RCCs. The LASSO, SVM, and decision tree model were conducted based on ‘glmnet’, ‘e1071’, and ‘rpart’ packages, respectively. The receiver operating characteristics (ROC) curve analysis was performed to determine the AUC, accuracy, specificity and sensitivity for evaluating the performance of the model. The significance was set at *P* < 0.05.

## Results

**Figure 2:**
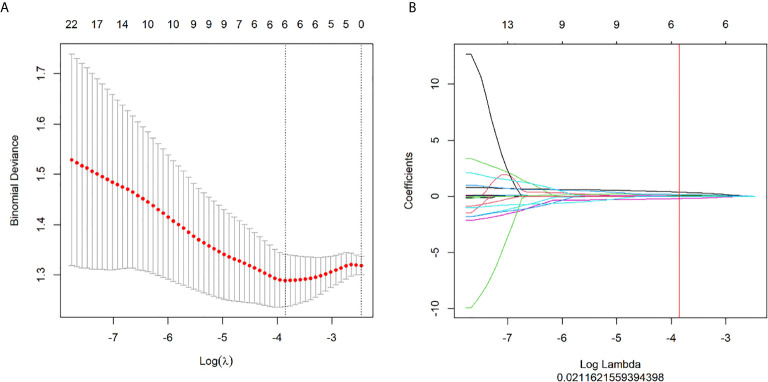
Feature selection with the LASSO method. **(A)** The tuning parameter (λ) changes in the LASSO model. The binomial deviance curve was generated with the log (λ). The minimum criteria for five-fold cross-validation were applied. The best λ = 0.0212 was obtained at the minimal binomial deviance. **(B)** The LASSO coefficient profile plot with different log (λ) was shown. The vertical red line was the best λ with 6 selected radiomic features.

**Figure 3:**
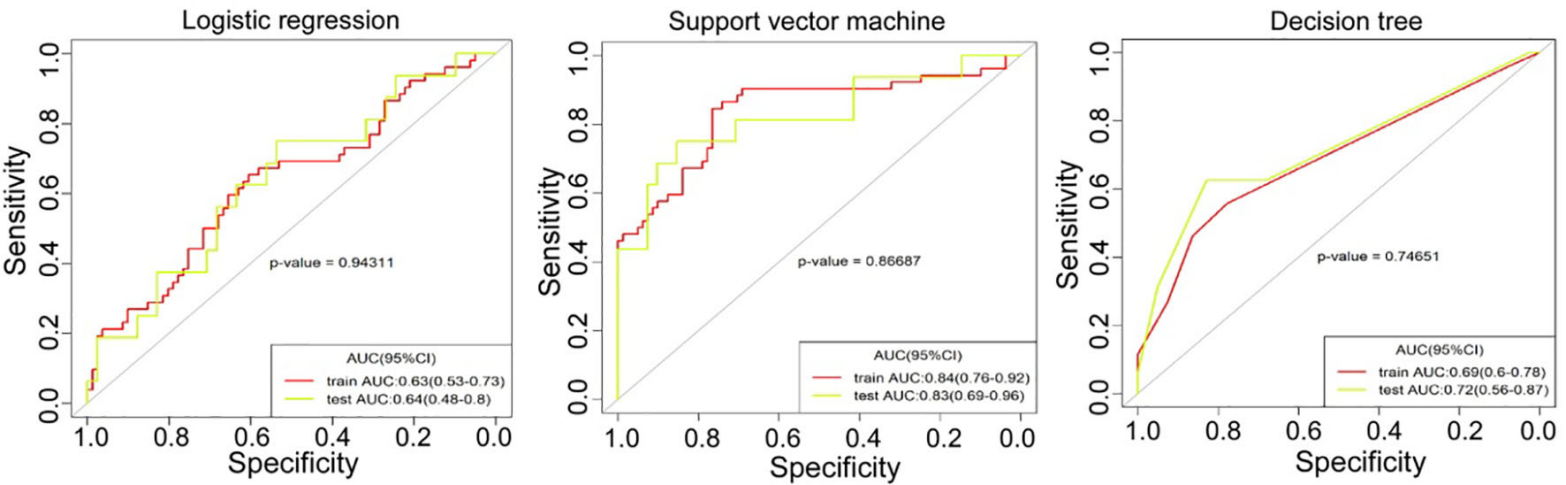
The receiver operating characteristics (ROC) curve analysis was performed for three models of logistic regression, support vector machine and decision tree in the training set and testing set.

The six most valuable features selected by LASSO for radiomics modelling were GLCMEntropy, GreyLevelNonuniformity, ShortRunEmphasis, LongRunLowGreyLevelEmphasis, ShortRunLowGreyLevelEmphasis, and IntensityVariability. The LASSO regression was shown in **Figure 2**. The specific parameters and feature extraction used in the six most valuable features were demonstrated in **Table 2**. These features were used to establish three models of logistic regression, decision tree and SVM in the training set with 122 patients. Each model was trained and assessed using the repeated ten-fold cross-validation method in the training set. Performance of differentiating high grade from low grade ccRCC was evaluated with the testing set (68 patients) (**Figure 3**).

**Table 2:** Specific parameters and feature extraction in six features.

ID	Class	Type	Offset	Direction
1	GLCM	Entropy	7	Angle90
2	RLM	GreyLevelNonuniformity	7	All (3D)
3	RLM	ShortRunEmphasis	7	Angle0
4	RLM	LongRunLowGreyLevelEmphasis	7	Angle0
5	RLM	ShortRunLowGreyLevelEmphasis	4	All (3D)
6	Histogram	IntensityVariability	–	–

The AUC values in the training set and testing sets are respectively 0.63 (95% CI 0.53-0.73) and 0.64 (95% CI 0.48-0.8) with the logistic regression model, 0.84 (95% CI 0.76-0.92) and 0.83 (95% CI 0.69-0.96) with SVM model, and finally, 0.69 (95% CI 0.60-0.78) and 0.72 (95% CI 0.56-0.87) with the decision tree model. The cutoff value of each model was obtained from the Youden index from the ROC curve, with the value being 0.366, 0.38, and 0.276, respectively, in the logistic regression, SVM, and decision tree for the test set. The results presented in **Tables 3** and **4** showed that the SVM model had achieved the best performance.

**Table 3:** ROC curve analysis of three models in the training set.

Parameter	Logistic (Train)	SVM (Train)	Decision Tree (Train)
AUC	0.632 (CI: 0.533–0.730)	0.840 (CI: 0.653–0.758)	0.688 (CI: 0.601–0.775)
Accuracy	0.624 (CI: 0.530–0.707)	0.797 (CI: 0.719–0.862)	0.692 (CI: 0.606–0.769)
Sensitivity	0.654 (CI: 0.462–0.788)	0.846 (CI: 0.558–0.942)	0.558 (CI: 0.385–0.681)
Specificity	0.605 (CI: 0.272–0.741)	0.742 (CI: 0.284–0.852)	0.778 (CI: 0.575–0.904)

ROC, Receiver operating characteristic; AUC, area under the operating curve; CI, confidence interval.

**Table 4:** ROC curve analysis of three models in the testing set.

Parameter	Logistic regression	Support vector machine	Decision Tree
AUC	0.639 (CI: 0.476–0.802)	0.826 (CI: 0.688–0.964)	0.717 (CI: 0.564–0.871)
Accuracy	0.596 (CI: 0.458–0.724)	0.825 (CI: 0.701–0.913)	0.772 (CI: 0.642–0.873)
Sensitivity	0.750 (CI: 0.436–0.938)	0.750 (CI: 0.438–0.938)	0.625 (CI: 0.320–0.812)
Specificity	0.537 (CI: 0.195–0.756)	0.854 (CI: 0.341–0.976)	0.829 (CI: 0.400–0.951)

ROC, Receiver operating characteristic; AUC, area under the operating curve; CI, confidence interval.

**Figure 4:**
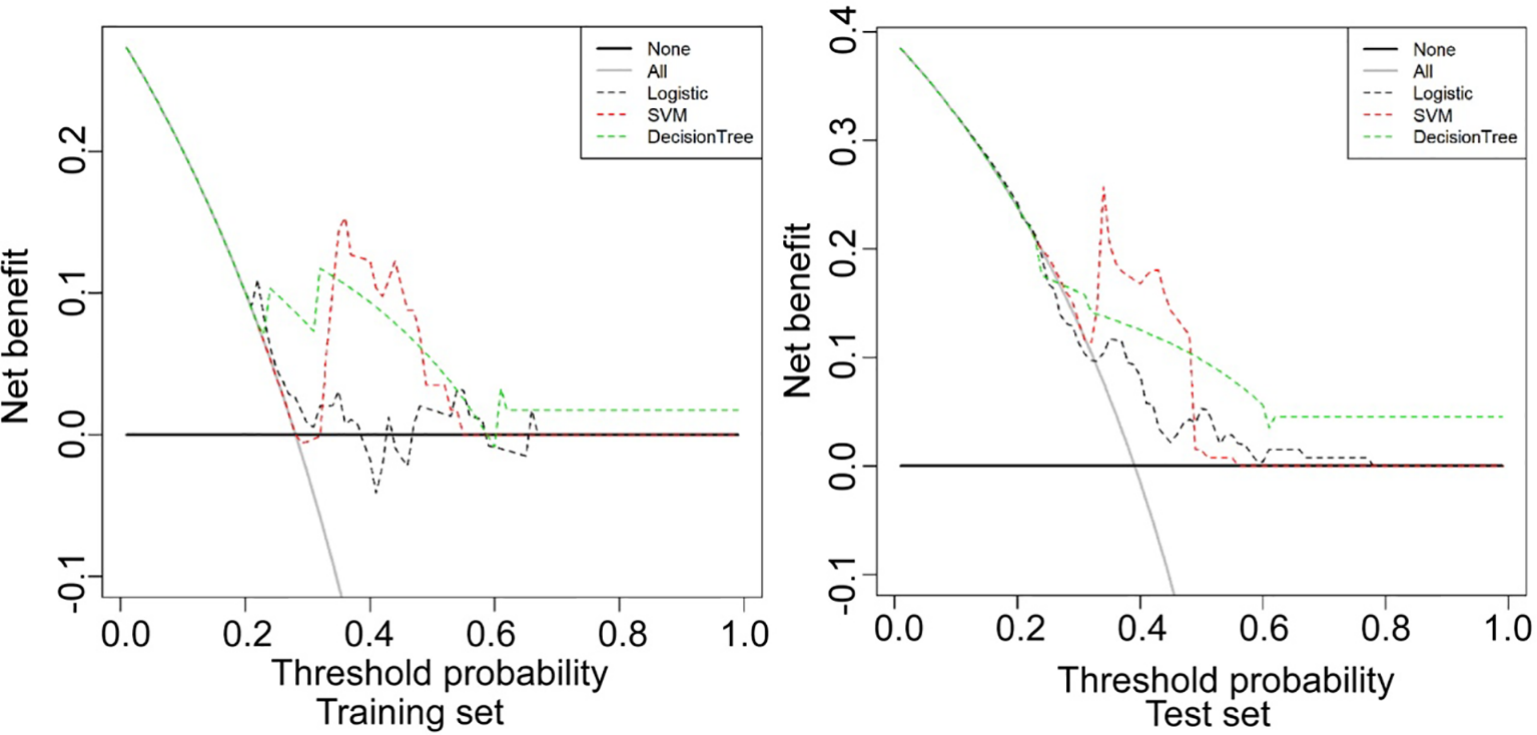
Decision curve analysis (DCA) was conducted to evaluate the clinical usefulness of the models in prediction by quantifying the net benefits at different threshold probabilities in the training and testing set. The SVM model had the best performance in prediction of low- and high-grade renal cell carcinoma. Logistic, logistic regression model; SVM, support vector machine model; Decision tree, decision tree model.

DCA was conducted to evaluate clinical usefulness of the models in prediction by quantifying the net benefits (relative benefits), at different threshold probabilities in both sets (**Figure 4**). The SVM model had the best performance in prediction of low- and high- grade renal cell carcinoma. In the DCA analysis (**Figure 4**), the SVM model was shown to obtain the highest benefit in the range of 0.34-0.49 which contained the cutoff value 0.38 for the SVM model. The “benefit” was relative and indicated the efficiency of the models in the test set.

**Figure 5:**
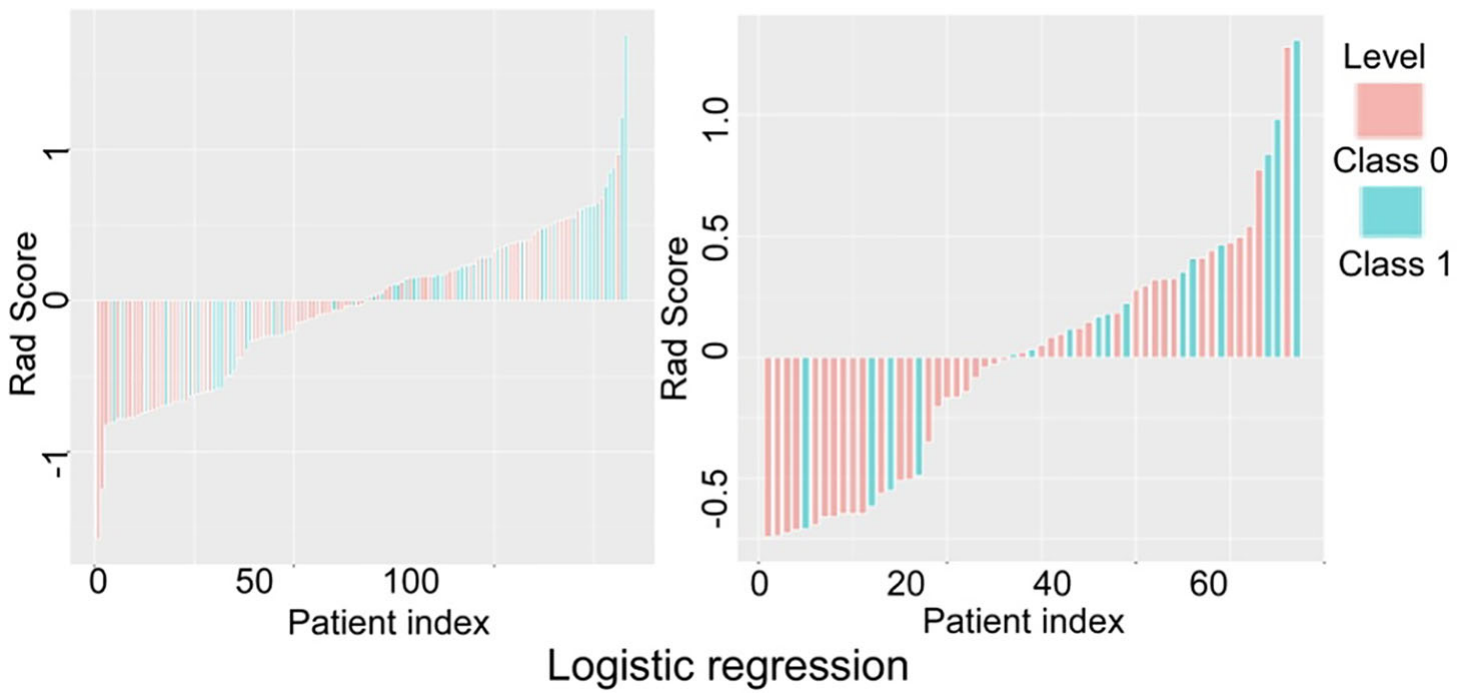
Verification and comparison of the logistic regression model in predicting low and high grade renal cell carcinoma in the training and testing set. In the training set, the true negative rate (specificity) for predicting grade I-II clear cell renal cell carcinoma (ccRCC) was 60.5% (49/81), and the true positive rate (sensitivity) for predicting grade III-IV ccRCC was 65.4% (34/52). In the testing set, the true negative rate was 53.7% (22/41) for predicting grade I-II ccRCC and 75% (12/16) for predicting grade III-IV.

**Figure 6:**
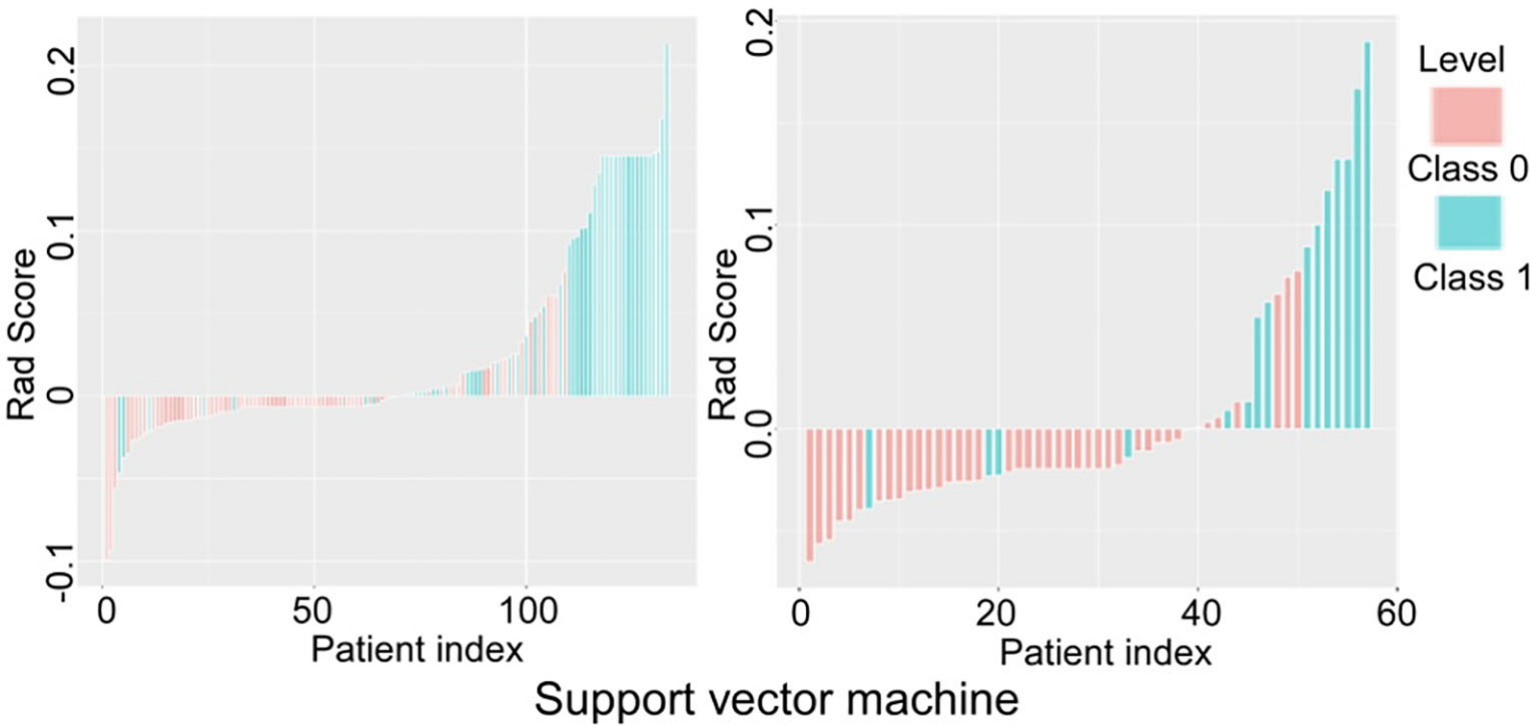
Verification and comparison of the support vector machine model in predicting low and high grade renal cell carcinoma in the training and testing set. In the training set, the true negative rate (specificity) was 76.5% (62/81) for predicting grade I-II clear cell renal cell carcinoma (ccRCC), and the true positive rate (sensitivity) was 84.6% (44/52). In the testing set, the true negative rate was 85.4% (35/41) for predicting grade I-II ccRCC and 75% (12/16) for predicting grade III-IV ccRCC.

**Figure 7:**
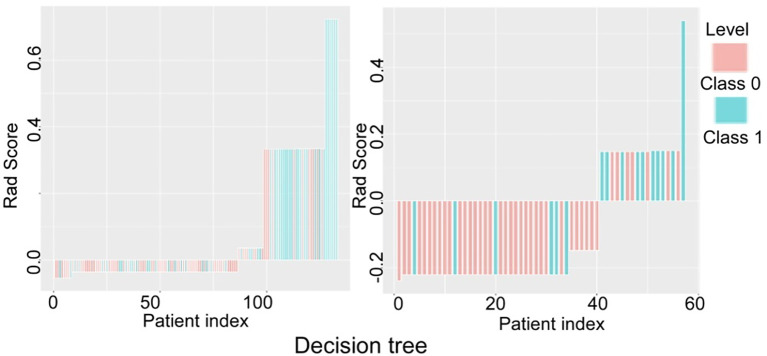
Verification and comparison of the decision tree model in predicting low and high grade renal cell carcinoma in the training and testing set. In the training set, the true negative rate (specificity) was 77.8% (63/81) for predicting grade I-II clear cell renal cell carcinoma (ccRCC), and the true positive rate (sensitivity) was 55.8% (29/52). In the testing set, the true negative rate was 82.9% (34/41) for predicting grade I-II ccRCC and 62.5% (10/16) for predicting grade III-IV ccRCC.

The prediction performance of the three models for low and high grade RCC was verified and compared (**Figures 5**–**7**). There was no significant (*P*=0.054) difference in the high and low-grade distribution of ccRCCs between the training and testing sets.

In verification of the logistic regression model (**Figure 5**), the true negative rate (specificity) for predicting grade I-II ccRCC was 60.5% (49/81) in the training and 53.7% (22/41) in the testing set, and the true positive rate (sensitivity) for predicting grade III-IV ccRCC was 65.4% (34/52) in the training and 75% (12/16) in the testing set. In verification of the SVM model (**Figure 6**), the true negative rate (specificity) was 76.5% (62/81) for predicting grade I-II ccRCC in the training and 85.4% (35/41) in the testing set, and the true positive rate (sensitivity) was 84.6% (44/52) in the training set. The testing set also exhibited a true negative rate of 75% (12/16) for predicting grade III-IV ccRCC. In verification of the decision tree model (**Figure 7**), the true negative rate (specificity) was 77.8% (63/81) for predicting grade I-II ccRCC with the true positive rate (sensitivity) of 55.8% (29/52) in the training set. For the testing set, the true negative rate was 82.9% (34/41) for predicting grade I-II ccRCC and 62.5% (10/16) for predicting grade III-IV ccRCC.

The AUC, accuracy, specificity, and sensitivity were used to evaluate the performance of the model (**Tables 1** and **2**). Through comprehensive comparison of the AUC value, specificity, sensitivity of the three models, the best prediction efficiency, observed in the sSVM model, was therefore selected for prediction purpose in this study. The SVM model had the greatest accuracy (0.797 and 0.825), sensitivity (0.846 and 0.825) and specificity (0.742 and 0.750) in both the training and testing set compared with the logistic regression model (0.624 and 0.596, 0.654 and 0.750, 0.605 and 0.537, respectively) and the decision tree model (0.692 and 0.772, 0.558 and 0.625, 0.778 and 0.829, respectively).

## Discussion

The present study was aimed at the differentiation of high- from low-grade ccRCCs, because pathological grades highly correlate with ccRCC metastasis and prognosis (20). ccRCC has different clinical prognoses at different grades, and early identification of pathological grade of ccRCCs is valuable for timely clinical treatment and patient health.

Radiomics analysis is to extract a multitude of features form medical images to analyze size, shape, and texture, with useful spatial information on pixel or voxel distribution and modes. The recent advancements in the study of ccRCCs were based on imaging histology except for its grading (21). In the modeling and identification of high- and low-grade ccRCCs, previous studies (22, 23) used *in vivo* diffusion-weighted imaging (DWI) and imaging histology to achieve the AUC value of 0.8, whereas an AUC value of 0.73 was reached by the Renometric score based on CT imaging in identification of high-level RCCs (23). The AUC values for SVM model in the training and testing sets in our study were 0.84 and 0.83, respectively, higher than 0.8 or 0.73 of methods described earlier.

Ding et al. (13) applied radiomics to establish three logistic regression models to identify high and low-grade ccRCCs, achieving the AUC values in the training sets of the three models of 0.826, 0.878, 0.843 compared with the AUC values in the testing sets of 0.671, 0.771 and 0.780, respectively. Although the results in training set were better, the scores in testing set were not as satisfactory probably due to a trend of over-fitting. In addition, Ding et al. extracted the texture features from the maximal diameter level of the mass and collected less heterogeneous information of the mass (13). Compared with the study by Ding et al, our SVM-based model performed better, with our SVM-based AUC in the training and testing set being 0.84 and 0.83, respectively. Shu et al. (24) established three radiomic models based on renal CT enhancement images in the cortical and parenchymal phases, including cortical phase model, parenchymal phase model, and in combination. The corresponding accuracy, AUC value, sensitivity and specificity were 0.719, 0.766, 0.818 and 0.822) for the cortical phase model, 0.738, 0.602, 0.693 and 0.677 for the parenchymal phase model, and 0.777, 0.838, 0.838 and 0.839 for the combined model. Comparing these results to the study with 3D texture analysis based model by Shu et al. (24), our results have better accuracy, AUC value, and sensitivity. Although the model produced by Shu et al. (24) possessed slightly higher specificity with the combined multi-period model outperforming the one-period model, their study used full data to build the model without using independent test data to validate their results.

Radiomics-based grading models demonstrated better performance than the model based on conventional CT parameters. Chen et al. (14) pointed out that tumor size (TS) and permeability surface-area products (PS) were helpful in distinguishing the high and low grade clear cell renal cancers, with the AUC of both TS and PS being 0.7 and the sensitivity and specificity being 0.8 and 0.6 for TS and 0.7 and 0.8) for PS. The grading performance in our study was also better than this study (15).

Heterogeneity is an important feature of malignant tumors and is closely related to their biological behavior. CT enhanced imaging can be used to effectively evaluate tumor heterogeneity (25). After studying low enhancement on multiphase contrast-enhanced CT images for predicting presence of high tumor grade of ccRCC (26), Miles et al. found that low tumor enhancement in the cortico-medullary phases was an independent predictor of high tumor grade, which may be useful in clinical care of patients with nonsurgical approaches. It is speculated that the higher the grade of renal clear cell carcinoma, the more abundant the small capillaries (27), which is supported by another study by Li et al (15). In addition, necrosis is highly correlated with heterogeneity of tumors, which is of great significance (28). In this study (28), various processing techniques including voxel normalization and various filtering processes were used to extract a variety of high and low order features, including gray matrix and 3D morphological features. Finally, LASSO cross-processing was used to select the most valuable six histological features.

After looking into a variety of common first-order features that reflect tumor heterogeneity, such as average gray level, kurtosis and entropy, Feng et al. proposed that entropy is an independent and excellent radiomic feature to describe a degree of disorder in images (29). In terms of lesion density distribution, larger entropy values suggest more randomness while smaller entropy values indicate uniformity. Thus, high-grade tumors with relatively large liquefaction necrosis volume have reduced the entropy detectable as a radiomic feature and were consequently excluded from our study. In our study, we only studied the primary renal cell carcinoma rather than metastatic carcinomas from other resources. However, if the renal cancerous lesions of the primary renal cell carcinoma contained large-area necrosis or cystic changes, they would be excluded from the study, because necrosis contained inactive tissue and cystic changes contained liquid materials. Solid mass should be retained as much as possible. The radiomics captured tissues primarily with active and biological behavior, namely solid mass tissues. Cystic degeneration and necrosis are similar in nature, and the doping of these changes in the samples may lower the evaluation efficiency of the results.

In our study, GLCM_entropy, Greylevel_Nonuniformity, and Intensity_Variability of the six features reflect the degree of random gray distribution in ROI, which is usually used to demonstrate the tumor heterogeneity. ShortRun_Emphasis and ShortRunLowGreyLevel_Emphasis are used to show the fine texture of the tumor, whereas LongRunLowGreyLevel_Emphasis is used to reflect the coarse texture within the tumor. The SVM model in our study used the RBF kernel with C value 1 and gamma 0.001. The SVM is a nonlinear model which can get greater and better results than the linear model. The SVM model may be used for machine learning with small samples, for improving generalization and solving higher-dimensional problems as well as for avoiding structural selection in neural networks. There are some limitations in our study. Firstly, the overall sample size was relatively small. Secondly, patient data was not comprehensively collected, with the construction of models having excluded diagnostic elements from biochemistry, immunohistochemistry and genetic studies. Thirdly, when the VOI was delineated, the accuracy of the delineated lesions was reduced, due to unclear margins of some tumor masses or the influences by partial volume effect. Fourthly, the current single-center study lacked independent validation and evaluation from external professionals. Although our scanning parameters and reconstruction methods had been standardized, they should have been fixed with multicenter studies, thus necessitating a unified measurement standard for obtaining necessary information. Lastly, this study was limited to its retrospective nature and involvement with only Chinese ethnicity.

In summary, the current study uses radiomics analysis to differentiate the grade of ccRCC, and the support vector machine-based model exhibits the best performance for differentiating high- and low-grade ccRCC when compared to the logistic regression model and the decision tree model.

## Data Availability Statement

The raw data supporting the conclusions of this article will be made available by the authors, without undue reservation.

## Ethics Statement

The studies involving human participants were reviewed and approved by Ethics committee of Affiliated Hospital of Hebei University. The patients/participants provided their written informed consent to participate in this study.

## Author Contributions 

Study design: XP and J-LR. Data collection: XP, PW, L-YM, YW, and XM. Data analysis: XP, PW, J-LR, X-PY, and B-LG. Supervision: L-YM. Manuscript writing: XP. Revision: B-LG. All authors contributed to the article and approved the submitted version.

## Conflict of Interest

Author JR was employed by GE Healthcare company.

The remaining authors declare that the research was conducted in the absence of any commercial or financial relationships that could be construed as a potential conflict of interest.
